# Meta-Analysis of Seroprevalence and Prevalence of Influenza A Viruses (Subtypes H3N2, H3N8, and H1N1) in Dogs

**DOI:** 10.3390/ani14233467

**Published:** 2024-12-01

**Authors:** Ivan Ramos-Martínez, Edgar Ramos-Martínez, Manuel Saavedra-Montañez, Saraí Santos-Paniagua, René Segura-Velázquez, Onasis Vicente-Fermín, Mayte Martínez-Aguirre, Juan C. Ramos-Martínez, Sheila Irais Peña-Corona, Rodolfo Pastelin-Palacios, José Ivan Sánchez-Betancourt

**Affiliations:** 1 Departamento de Medicina y Zootecnia de Cerdos, Facultad de Medicina Veterinaria y Zootecnia, Universidad Nacional Autónoma de México, Ciudad de México 04510, Mexico; iramos.martinez88@gmail.com; 2 Escuela de Ciencias, Universidad Autónoma Benito Juárez de Oaxaca, Oaxaca 68110, Mexico; edgargus2@gmail.com; 3 Departamento de Microbiología e Inmunología, Facultad de Medicina Veterinaria y Zootecnia, Universidad Nacional Autónoma de México, Ciudad de México 04510, Mexico; manuelsaavedra76@gmail.com; 4 Unidad de Investigación, Facultad de Medicina Veterinaria y Zootecnia, Universidad Nacional Autónoma de México, Ciudad de México 04510, Mexico; santosp.sarai@gmail.com (S.S.-P.); realselab@gmail.com (R.S.-V.); 5 Escuela de Enfermería, Instituto Nacional de cardiología “Ignacio Chávez”, Ciudad de México 14080, Mexico; onasis.vicente.fermin@gmail.com; 6 Instituto de Química, Universidad Nacional Autónoma de Mexico, Ciudad de México 04510, Mexico; etyma@hotmail.com; 7 Departamento de Cardiología, Hospital General Regional “Lic Ignacio Garcia Tellez” IMSS, Mérida 97150, Mexico; dr.juancarlosramos@hotmail.com; 8 Departamento de Farmacia, Facultad de Química, Universidad Nacional Autónoma de Mexico, Ciudad de México 04510, Mexico; sheila.ipc@live.com; 9 Departamento de Biología, Facultad de Química, Universidad Nacional Autónoma de México, Ciudad de México 04510, Mexico; rodolfop@unam.mx

**Keywords:** influenza A, seroprevalence, prevalence, meta-analysis, dogs

## Abstract

Influenza A is a zoonotic disease that affects dogs, pigs, horses, poultry, and birds. In this report, a meta-analysis according to PRISMA (Preferred Reporting Items for Systematic Reviews and Meta-Analyses) was conducted. Studies of influenza A viruses in dogs providing prevalence or seroprevalence in any location worldwide were included. The results in this study show that the seroprevalence for the H3N8 influenza subtype was 7.96% (95% CI: 2.03–16.8, *p* < 0.001), for the H3N2 subtype was 7.44% (95% CI: 4.51–10.5, *p* < 0.001), and for the H1N1 subtype was 3.10% (95% CI: 0.890–6.01, *p* < 0.001). In the case of the prevalence analysis, a prevalence of 0.395% (95% CI: 0.160–2.44) for the H3N8 subtype. For the H3N2 subtype, a prevalence of 17.8% (95% CI: 6.66–32.6, *p* < 0.001) was found. No publication bias was observed in the studies evaluating seroprevalence in the H3N2 and H1N1 subtypes. In the H3N8 subtype, Begg’s test indicated publication bias, but Egger’s test showed no bias. It is essential to know the approximate prevalence and seroprevalence worldwide of canine influenza, so this study reports the presence of influenza subtypes in dogs, placing this species as a reservoir for human, swine, equine, and avian influenza A viruses.

## Simple Summary

Influenza A is a zoonotic disease that affects dogs. In this work, we performed a meta-analysis of the prevalence and seroprevalence of reports from all over the world. We found a combined seroprevalence of 7.6% for subtype H3N8, 7.44% for subtype H3N2, and 3.10% for subtype H1N1. Prevalence values were 0.395% for subtype H3N8 and 17.8% for subtype H3N2. The studies that evaluated seroprevalence in the H3N2 and H1N1 subtypes showed no publication bias, while the H3N8 subtype showed bias in one test and not in another. Dogs, as carriers of influenza A, are in direct contact with humans, pigs, equines, and birds; however, so far it is unknown whether they can function as a reservoir of the influenza A virus, so more studies are needed, with larger sample sizes and a wider distribution throughout the world. Knowing the prevalence and seroprevalence values in dogs is important in the creation of surveillance systems that allow the prevention of outbreaks.

## 1. Introduction

Influenza is a respiratory disease caused by influenza viruses that belong to the family Orthomyxoviridae; four types are known: Influenza virus types A, B, C, and D, according to the latest official nomenclature published by the “International Committee on Taxonomy of Viruses” [1]. Type A influenza viruses circulate in a wide range of species, including humans and domestic animals: dogs, pigs, horses, poultry, and wild migratory birds [2]. The defining event for the onset of canine influenza was the isolation of an influenza virus from racing greyhounds experiencing respiratory infections in early 2004 [3]. Currently, canine influenza causes endemic infections in different countries [4].

Influenza A viruses (IAV) contain a segmented negative-sense single-stranded RNA genome which consists of eight segments encoding up to 15 proteins, the virion possesses a lipid envelope that contains two major spike glycoproteins, hemagglutinin (HA) and neuraminidase (NA) [5]. On the basis of antigenic criteria for HA and NA, influenza A viruses are currently divided into 18 HA (H1 to H18) and 11 NA (N1 to N11) subtypes [6].

The ecology and epidemiology of IAV are very complex, and the emergence of novel zoonotic pathogens is one of the greatest challenges in healthcare. Zoonotic infections can stably adapt to humans, leading to sustained person-to-person transmission as in the case of IAVs. However, the mechanisms by which viruses stably adapt to new host species, often distantly related, are still largely unelucidated. [4]. The consensus from the literature indicates that dogs are reservoirs of influenza A and play an important role in the transmission and sustainability of the influenza A virus through contact with humans [7,8,9]. One Health High-Level Expert Panel (OHHLEP) has defined the elements of health as tracking all sectors, creating solutions that address the root causes and linkages between risks and outcomes that enable control and containment of disease risks. The “One Health” perspective prioritizes human health and the avoidance of diseases that can be transmitted from animals to humans (Tripartite and UNEP Support OHHLEP’s Definition of “One Health,” n.d.). The increasing number of canine influenza cases suggests that the One Health approach involving various public and private sector stakeholders would be a better option to successfully control and limit canine influenza outbreaks in various countries [10,11,12].

This meta-analysis aims to bring together the results of peer-reviewed observational studies reporting the prevalence or seroprevalence of different subtypes of Influenza A in dogs worldwide. Although there are meta-analyses of prevalence in humans and other species, so far there are no meta-analyses for dogs, which are one of the animals with which humans have the most significant contact [13]. Therefore, it is necessary to have a general estimation of the infection for the most common subtypes H3N8, H3N2, and H1N1 that infect dogs worldwide.

## 2. Materials and Methods

### 2.1. Protocol and Search Strategy

This meta-analysis was performed in accordance with the guidelines of PRISMA (Preferred Reporting Items for Systematic Reviews and Meta-Analyses) [14]. The search was conducted from March 2023 to June 2023 in PubMed, Web Science database, OVID-EMBASE, Scope, and EBSCOhost databases, and search engines such as Google Scholar. The following combined terms [(Influenza A [All Fields]) AND (Dogs OR canine [All Fields])] were searched. Two coauthors independently reviewed the abstracts generated by the search, then selected studies that would fit the inclusion criteria and read the full text to identify eligible studies. Some studies were identified by searching reference lists of relevant reviews and original articles. Studies in English and Spanish with full access to the article were included, and no restriction on the publication period was applied.

### 2.2. Eligibility Criteria

Inclusion criteria for this meta-analysis were (a) providing prevalence or seroprevalence of influenza A virus in dogs; (b) studies conducted in any location worldwide; (c) studies that reported the sample size and the number of positive samples; (d) studies that used tests for the presence of virus (PCR or virus isolation) or presence of antibody (hemagglutination inhibition or microneutralization). Exclusion criteria were: (a) duplicate studies of the original data; (b) articles were abstracts, commentaries, case reports, unpublished data, letters, reviews, or non-clinical studies; (c) there was no access to the full text; (d) infections were experimental.

### 2.3. Risk of Bias Assessment

Study quality was assessed by two independent reviewers, using the critical appraisal tools for the prevalence of the Joanna Briggs Institute (JBI). The studies were classified as low, high, or uncertain risk of bias based on a verification questionnaire [15].

### 2.4. Data Extraction

Data were extracted into an Excel sheet. The data extracted from the selected articles included the name of the first author, date of publication, location where the sample was obtained, detection method, sample size, number of positive cases, and virus subtype. Two reviewers extracted data independently; discrepancies were identified and resolved by discussion with a third reviewer.

### 2.5. Statistical Analysis

Statistical analysis in this meta-analysis was performed using Jamovi software (Version 2.3). The number of positive cases and the total number of cases were obtained for each study. Prevalence values were transformed using the Freeman-Tukey double arcsine transformed proportion [16]. To calculate the pooled proportion and its 95% confidence interval. These values were then retransformed to proportions with the inverse formula given by Miller [17].

The I^2^ test and the χ^2^-based Q test were used to analyze statistical heterogeneity between studies. When there was significant heterogeneity (I2 > 50% or *p* < 0.05) in the included studies, the random-effects model was calculated according to the DerSimonian-Laird method [18]. Otherwise, the fixed effects model was used [19]. Sensitivity analyses were also performed, in which one study at a time was eliminated to assess its impact on the meta-analysis results. Finally, bias analysis was performed using the funnel plot and Egger and Begg tests.

## 3. Results

### 3.1. Characteristics of the Selected Studies

**Figure 1:**
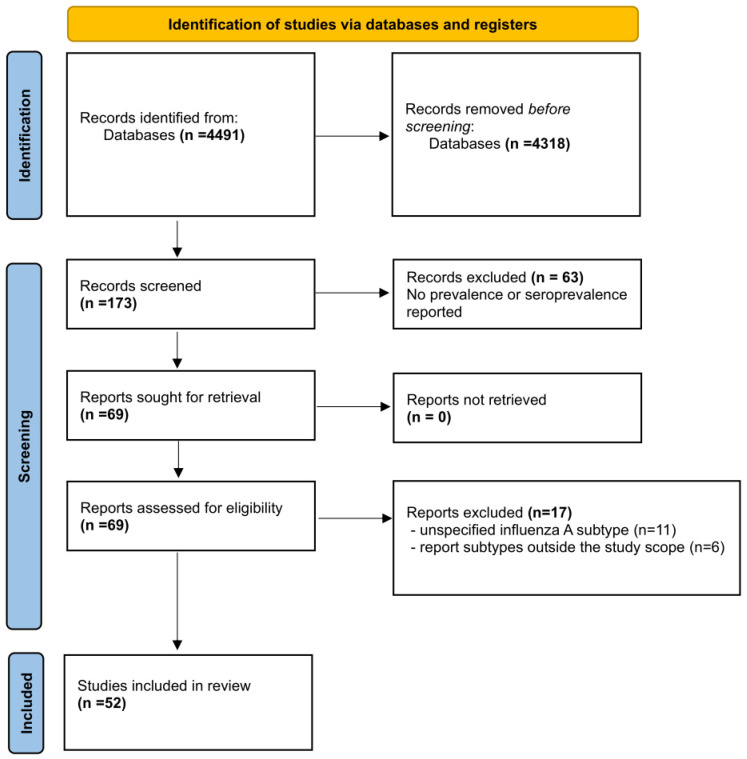
PRISMA flow diagram of the studies used in the current analysis.

A total of 4491 studies were found using the search strategy described above. A total of 4318 articles were eliminated because they were duplicates or not related to the object of study. The reviewers evaluated the title and abstract of 173 articles; 104 articles that were experimental infections or did not report influenza A prevalence/seroprevalence were excluded. The full text of 69 articles was reviewed, of which 17 articles were excluded, 11 because they did not specify the influenza A subtypes, and 6 because they reported other subtypes that were not within the scope of this meta-analysis (H5N2, H9N2, H10N8). Fifty-two articles were included in the quantitative analysis (some reported prevalence/seroprevalence of more than one subtype of interest). Of the H3N2 subtype, there were 31 reports; for H3N8, there were 19 reports; and for H1N1, there were 14 reports. These studies together included a sample of 9512 dogs for H3N8, 37,578 dogs for H3N2, and 10,330 dogs for H1N1 (Figure 1).

Regarding the distribution of the studies, 24 studies were conducted in East Asia, 18 in North America, 7 in Europe, 2 in Africa, and 1 in Oceania. Most of the studies came from samples from China (16 studies) and the United States of America (13 studies). Of the included studies, 42 reported seroprevalence and 15 prevalence. Regarding the detection method, the hemagglutination inhibition (HI) assay was used for seroprevalence in 40 studies and the microneutralization (MN) assay in 2 studies; for prevalence, 15 studies used RT-PCR (Table 1, Table 2 and Table 3).

### 3.2. Risk of Bias Assessment

**Figure 2:**
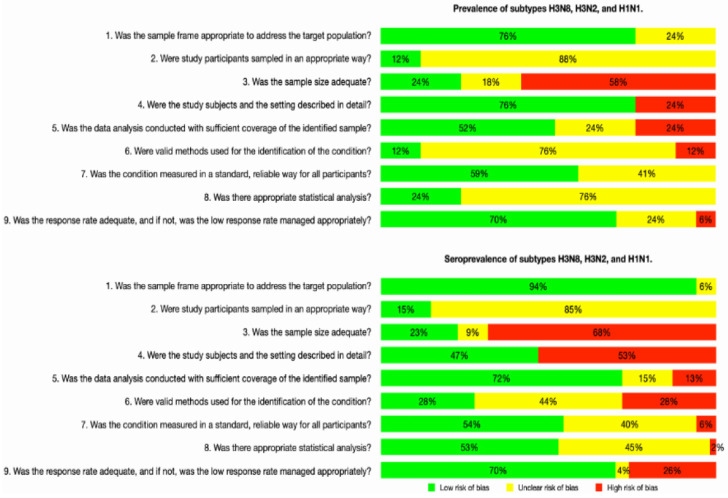
Risk-of-bias assessment of eligible studies on prevalence and seroprevalence of canine influenza viruses (subtypes H3N8, H3N2, and H1N1), using the Joanna Briggs Institute (JBI) critical appraisal tool for prevalence studies.

The quality of the studies was established with the Joanna Briggs Institute (JBI) critical appraisal tool for prevalence studies. Each study result was classified and evaluated according to a verification questionnaire to determine whether it had a low, uncertain, or high-risk bias (Figure 2). Each report result was classified and evaluated according to a verification questionnaire.

76% of the prevalence studies and 94% of the seroprevalence studies had a low-risk bias because the type of sample was adequate. In general, for studies related to prevalence and seroprevalence, it can be observed that most of the studies do not specify how the sample was taken from the population, so the risk of bias is not clear on this point.

Regarding the sample size, it was determined that the risk of bias was high, both for prevalence and seroprevalence, since it was considered that the adequate sample size should be at least 1000 samples, and only 24% and 23% of the articles consulted for prevalence and seroprevalence, respectively, met this criterion. On the other hand, in the description of the subjects under study and their environment, we can observe that the behavior of the prevalence and seroprevalence was very different since, for the first case, 76% of the articles analyzed presented a low bias, while for the second case, more than half of the articles analyzed, corresponding to 53%, presented a high bias. Another analyzed criterion was whether the statistical analysis involved the entire sample. In general, it was observed that in both prevalence and seroprevalence, the risk of bias was low, with a percentage of 52% and 72%, respectively.

In most of the studies analyzed, it is not clear whether they used statistical methods to reach a result and fulfill the established objective, so at this point, the risk bias is not clear for prevalence with 76% and for seroprevalence with 44%. All experiments detected antibodies in serum using appropriate methods, including the HI (40 studies) and MN assays (2 studies); therefore, a low-risk bias is considered at this point. Finally, in all studies, the number of tested and positive samples was clearly indicated, and therefore a low-risk bias was determined.

### 3.3. Seroprevalence and Prevalence of Influenza A Virus in Dogs

**Figure 3:**
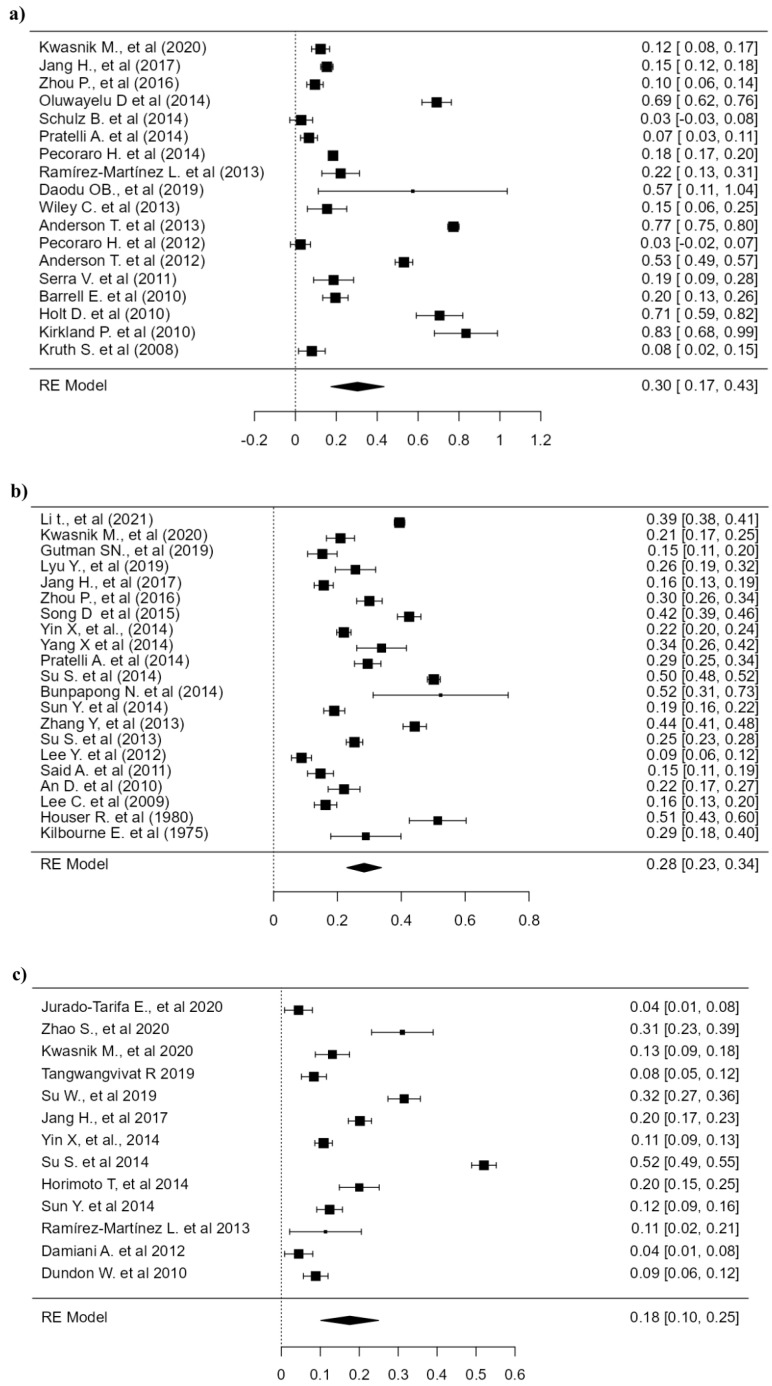
Forest plots of the seroprevalence of influenza A virus in dogs. (**a**) Meta-analysis of the seroprevalence of H3N8. (**b**) Meta-analysis of the seroprevalence of H3N2. (**c**) Meta-analysis of the seroprevalence of H1N1. Data are Freeman–Tukey double arcsine transformed proportions. CI 95%: 95% confidence interval; RE: random effect model.

The estimated pooled seroprevalence for the H3N8 influenza A subtype was 7.96% (95% CI: 2.03–16.8, *p* < 0.001), for the H3N2 subtype was 7.44% (95% CI: 4.51–10.5, *p* < 0.001), and for the H1N1 subtype was 3.10% (95% CI: 0.890–6.01, *p* < 0.001). All these analyses were performed with the random effects model since the heterogeneity was greater than 98%. The analysis results are shown in the forest plot (Figure 3).

In the case of the prevalence analysis, a prevalence of 0.395% (95% CI: 0.160–2.44) was found for the H3N8 subtype. However, it was not significant (*p* = 0.103), as only 3 studies were included, two of which reported zero prevalence in their samples (Table 1). For the H3N2 subtype, a prevalence of 17.8% (95% CI: 6.66–32.6, *p* < 0.001) was found. No meta-analysis was performed for the H1N1 subtype since only one study reported prevalence, results of the prevalence analysis are shown in Table 4.

The subgroup analysis was performed according to the location where the samples were taken; subgroup analysis was only performed when there were 3 or more studies in the same region. For the H3N8 subtype, a large difference was observed between seroprevalence in North America (7.96%) and seroprevalence in Europe (0.379%). For seroprevalence in Asia, Africa, and Oceania, there was only one study each, and the sample size was small (Table 1). For serotype H3N2, similar seroprevalence was observed between studies conducted in East Asia (7.97%) and studies conducted in North America (6.86%). For the European studies, there were only two studies, and therefore, no meta-analysis was performed, but the prevalence reported in these studies was similar to those found for East Asia and North America (Table 2). Regarding the prevalence of the H3N2 subtype, the values found were 16.2% for East Asia and 20.4% for North America. Finally, for the H1N1 subtype, a higher seroprevalence was observed in East Asia (5.136%) than in Europe (1.31%); for North America, only two studies have been carried out, both of which report low seroprevalences (Table 5).

### 3.4. Sensitivity Analysis and Publication Bias

Sensitivity analysis was performed by eliminating one study at a time to assess the individual impact on seroprevalence and prevalence estimate. The analysis shows that studies assessing seroprevalence in the H3N8 and H3N2 subtypes do not significantly affect the pooled prevalence estimate or its confidence interval (Supplementary Materials Figure S1a,b). In the case of the H1N1 subtype, the study by Su S. et al. (2014) [68] increases the prevalence and its confidence interval because it is a study with a large sample (*n* = 960) and reports a high prevalence (24.7%) (Supplementary Materials Figure S1c). In the case of the prevalence analysis for the H3N2 subtype, it was observed that the study by Zhou L. et al. (2019) [44] has a notable impact on the calculation of the prevalence and its confidence interval (Supplementary Materials Figure S1d), this because it reports a prevalence of 100% in its sample; however, given the small sample size of this study, its results should be taken with caution. Sensitivity analysis of the prevalence of the H3N8 subtype was not performed since the number of studies was less than required.

**Figure 4:**
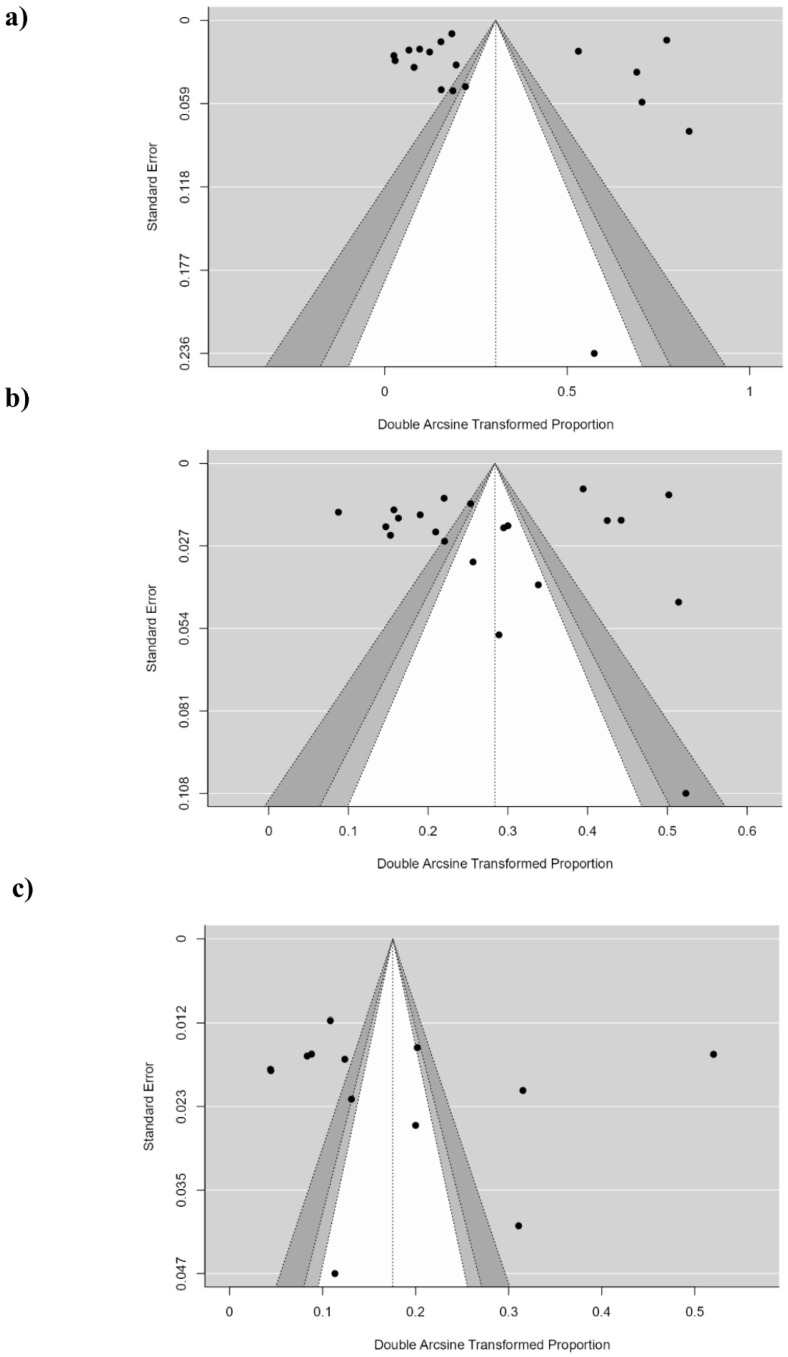
Publication bias and sensitivity analysis. (**a**) Funnel plot for the analysis of H3N8 seroprevalence in dogs. (**b**) Funnel plot for the analysis of H3N2 seroprevalence in dogs. (**c**) Funnel plot for the analysis of H1N1 seroprevalence in dogs.

Publication bias was explored using funnel plots and Egger and Begg tests. The funnel plots show the arcsine square root transformed proportion on the abscissa axis and the standard error on the ordinate axis. In the seroprevalence studies, a symmetrical distribution was observed in the H3N2 subtype and a slightly asymmetrical distribution in the studies of the H3N8 and H1N1 subtypes, indicating a possible publication bias (Figure 4). To check for bias, Egger’s test and Begg’s test were applied, which indicated that there is no publication bias for the H3N2 influenza A studies (Z-value = 1.597; *p* = 0.110 and Kendall’s Tau = 0.143; *p* = 0.386), nor for the H1N1 influenza A studies (Z-value = 0.334; *p* = 0.738 and Kendall’s Tau = 0.179; *p* = 0.435). In the case of the H3N8 subtype, Egger’s test indicated no publication bias (Z-value = 1.440; *p* = 0.150), but Begg’s test did indicate a possible publication bias (Kendall’s Tau = 0.359; *p* = 0.039).

The prevalence study funnel plots show asymmetric distributions for the H3N8 and H3N2 subtypes (Supplementary Materials Figure S2), which agrees with the results of Egger’s test. In the case of the H3N8 subtype, Begg’s test did not indicate bias (Kendall’s Tau = −0.333; *p* = 1.000), but Egger’s test did indicate a significant bias (Z-value = −6.136; *p* = 0.001). Something similar was observed for the H3N2 subtype, where Begg’s test showed no bias (Kendall’s Tau = 0.205; *p* = 0.367), but Egger’s test did (Z-value = 3.337; *p* < 0.001).

## 4. Discussion

Dogs are the most common companion animals in cities and rural communities, as they participate as caretakers of farm animals in the herding of livestock as well as in the hunting of wild animals near wetlands [72,73]. These activities place dogs as a fundamental piece in the transmission of zoonotic diseases, as they increase the risk of the transmission of zoonotic infectious agents by the interaction between wild and domestic animals and humans [74].

The presence of subtypes of human origin in dogs has been demonstrated since there are several reports of the presence of antibodies to H1N1 and H3N2 subtypes, as well as the achievement of viral isolation of the 2009 pandemic H1N1 subtype [64,66,70,71]. On the other hand, a new H3N1 subtype has been reported that presents rearrangements in the canine genes of the pandemic H1N1 virus of human origin and canine H3N2, which is highly relevant since it is not known with certainty whether this genetic rearrangement arose within the dog [75]. Both dogs and humans have glycoproteins with terminal sialic acid; these glycans serve as receptors for IAVs, increasing the zoonotic risk [76,77].

Of the criteria used in the risk of bias analysis, two presented the most significant risk of bias. The first was the sample size for both prevalence and seroprevalence. Regarding this point, we can say that small studies showed higher prevalence and seroprevalence rates than extensive studies. One possible reason is that the extensive studies included in the analysis are mostly government reports that collected samples in each country as part of a normal surveillance routine. This reduces the possibility of obtaining positive samples compared to small studies, which mainly collected samples from specific locations during or after an outbreak of canine influenza. This increased the probability of obtaining more positive samples. The second criterion was whether the study subjects were described in detail. In this case, the analysis showed a high bias in seroprevalence (question 6). This behavior may be because it was taken as a criterion that the study analyzed complied with the description of both elements and not only with one of them. Concerning the type of sample, this was the criterion that presented the least risk of bias in both the prevalence rate and seroprevalence. This reaffirms that for prevalence, nasal swabs, oral swabs, and/or lung tissue samples are the most recommended, while for seroprevalence, blood to obtain serum was performed adequately, according to the methodology consulted. Influenza A studies in dogs are few compared to studies in humans, pigs, or poultry; however, according to the results of sensitivity and publication bias in this study, it is recommended to carry out more studies in dogs that include a greater number of samples that allow detecting the real prevalence in cities as well as in rural areas. Another weakness is that of the 52 studies, the majority belonged to only two countries: China with 16 studies and the USA with 13, so surveillance studies in other countries are needed to achieve a more equitable distribution around the world.

More subtypes should be studied in dogs, including avian and equine viruses, because since 2004 the H3N8 subtype of equine origin, infected dogs showed respiratory clinical signs; later in 2008, the H3N2 subtype of avian origin was isolated, and in different studies with both subtypes were reported in dogs from different countries [3,51,78,79]. One more report in dogs is exposure to the avian H7 subtype and infection to the avian H5N2 subtype, which shows that dogs are also susceptible to infection by other avian viruses [75,80].

The pooled seroprevalence of 7.96% for subtype H3N8 suggests that dogs have been in contact with infected equines, while the pooled seroprevalence of 7.44% for subtype H3N2 is associated with contact with humans, pigs, and poultry. For this reason, the seroprevalence of both subtypes is linked to these four species. When the analysis was performed according to location, similar results were observed for subtype H3N2 between East Asia (7.97%) and North America (6.86%). In the other regions, there were not enough reports. For subtype H3N8, there was a considerable difference between North America (7.97%) and Europe (0.379%). The seroprevalence of the different viral subtypes is highly dependent on the species being monitored and the subtype to be identified. This is due to most studies reporting the detection of the viral subtype or the presence of antibodies of the subtype previously reported in each species. This bias reduces the chances of finding new subtypes in species such as the dog, which interacts with other species such as horses, poultry, pigs, rodents, or other species with the same habitat.

No publication bias was observed in the studies evaluating seroprevalence in the H3N2 and H1N1 subtypes. In the H3N8 subtype, Begg’s test indicated publication bias, but Egger’s test showed no bias. This discrepancy between test results may be because Begg’s test has low statistical power, so it is more sensitive to studies with small samples, while Egger’s test is more specific [68]. For the publication bias analysis for prevalence, bias was found in the studies of H3N8 subtypes, as the number of available studies is small (*n* = 3) and two of these studies reported a prevalence of zero. In the case of the H3N2 subtype, bias was found because most studies report low prevalence, but two studies report very high prevalence [43,44] of 39.5% and 100%. This causes asymmetry in the funnel plots.

Only four studies found dogs vaccinated for influenza A with low vaccination percentages: 2.7% [47], 6.25% [49], 9.7% [30], and 9.8% [27], indicating a low vaccination rate. Furthermore, considering that the exposure in pet dogs has been reported for equine H3N8, 2009 human pandemic H1N1, as well as seasonal human H1N1 and H3N2 subtypes [28], studies in other countries show that dogs can participate as hosts for zoonotic diseases [72], including pathogens such as canine rabies. Therefore, this systematic study addresses the presence of influenza A viruses in dogs as well as the reports described above, placing the dog as a potential reservoir of influenza A viruses in humans, swine, equine, and avian.

## 5. Conclusions

According to the results of this study, it is observed that, despite the high seroprevalence of influenza in dogs, zoonotic transmissions from dogs are rare. However, it is important to determine the zoonotic spread of Influenza A. The “One Health” approach includes the zoonotic pathway and its contribution. In this regard, it is necessary to determine the contribution of zoonotic pathways such as dog kennels, street environments, clinics, homes, and shelters where close contact with people and dogs is maintained during handling as these practices promote the risk of human-to-dog transmission of influenza A. Therefore, it is essential to know the approximate value worldwide of the prevalence and seroprevalence of canine influenza for use as an early warning system.

## Figures and Tables

**Table 1:** Characteristics of reports of subtype H3N8.

Reference	Location	Results		Sampling Year
		Prevalence (+/total; %)	Seroprevalence (+/total; %)	
[20]	Poland	-	7/496; 1.41%	2016–2017
[21]	United States	-	25/1082; 2.3%	2012–2014
[22]	China	0/420; 0%	-	2012–2015
[23]	China	-	5/600; 0.83%	2015
[24]	Nigeria	-	75/185; 40.5%	-
[25]	Germany	0/307; 0%	0/307; 0%	2010–2011
[26]	Italy	-	2/562; 0.35%	1997–2011
[27]	Canada	66/2775; 2.37%	92/2764; 3.3%	2009–2012
[28]	Mexico	-	5/113; 4%	2012
[29]	Nigeria	-	1/4; 25%	2014
[30]	United States	-	2/103; 1.9%	2009
[31]	United States	-	618/1268; 49%	2005–2009
[32]	Canada	-	0/399; 0%	2010
[33]	United States	-	133/520; 25.6%	1999–2004
[34]	United States	-	3/100; 3%	2009
[35]	United States	-	9/250; 3.6%	2009
[36]	United States	-	31/74; 42%	2008
[37]	Australia	-	22/40; 55%	2007
[38]	Canada	-	1/225; 0.4%	2007

**Table 2:** Characteristics of reports of subtype H3N2.

Reference	Location	Results		Year
		Prevalence (+/total; %)	Seroprevalence (+/total; %)	
[39]	China	235/4174; (5.63%)	-	2017–2019
[40]	China	11/180; 6.11%	-	2018–2021
[41]	China	-	528/3579; 14.75%	2016–2018
[42]	China	128/693; 18.51%	-	2019
[20]	Poland	-	21/496; 4.23%	2016–2017
[43]	Canada	104/263; 39.5%	-	2017–2018
[44]	China	17/17; 100%	-	2017
[45]	United States	-	10/452; 2.21%	2015
[46]	China	54/399; 13.5%	15/240; 6.3%	2012–2017
[47]	United States	40/120; 33%	-	2015
[48]	United States	1693/12,837; 13.2%	-	2015–2016
[21]	United States	-	26/1082; 2.4%	2012–2014
[22]	China	9/420; 2.1%	-	2012–2015
[23]	China	-	52/600; 8.66%	2015
[49]	United States	16/300; 5.3%	-	2015
[50]	China	35/261; 13.4%	-	2013
[51]	Korea	-	121/715; 16.9%	2007–2011
[52]	China	-	91/1920; 4.7%	2012
[53]	China	-	17/158; 10.8%	2010–2012
[26]	Italy	-	47/562; 8.36%	1997–2011
[54]	China	93/2357; 3.9%	545/2357; 23.1%	2011–2013
[55]	Thailand	3/21; 14.28%	5/21; 23.81%	2012
[56]	China	-	31/882; 3.5%	2012–2013
[57]	China	-	132/723; 18.3%	2012
[54]	China	-	90/1440; 6.25%	2011–2012
[58]	Korea	-	7/980; 0.7%	2004–2009
[59]	Japan	-	12/582; 2.1%	2002–2008
[60]	Korea	-	18/385; 4.7%	2008
[61]	Korea	-	20/780; 2.6%	2006
[62]	United States	-	29/121; 24%	1978
[63]	United States	-	6/79; 7.6%	1973

**Table 3:** Characteristics of reports of subtype H1N1.

Reference	Location	Results		Year
		Prevalence (+/total; %)	Seroprevalence (+/total; %)	
[64]	Spain	-	1/750; 0.13%	2013–2016
[65]	Netherlands	-	14/154; 9.1%	2019
[20]	Poland	-	8/496; 1.61%	2019
[66]	Thailand	-	6/932; 0.64%	2011–2014
[67]	Hong Kong	-	53/555; 9.5%	2015–2017
[21]	United States	-	43/1082; 4%	2012–2014
[22]	China	0/420; 0%	-	2012–2015
[52]	China	-	22/1920; 1.1%	2012
[68]	China	-	237/960; 24.7%	2012
[69]	Japan	-	14/366; 3.8%	2009–2010
[56]	China	-	13/882; 1.5%	2012–2013
[28]	Mexico	-	1/113; 0.9%	2012
[70]	Germany	-	1/736; 0.13%	2010–2011
[71]	Italy	-	7/964; 0.7%	2009

**Table 4:** Results of the meta-analysis of the prevalence of influenza virus subtypes H3N8 and H3N2 in dogs.

Subtype (*n* = No. of Reports)	Prevalence	IC 95%	*p*	Heterogeneity (I^2^)	Model	Bias Beggs	Bias Eggers
H3N8 (*n* = 3)	0.39	[0.16–2.4]	0.103	93%	RM	Kendall’s Tau = −0.333; *p* = 1.000	Valor z = −6.136; *p* = 0.001
H3N2 (*n* = 13)	17.8	[6.66–32.6]	0.001	99%	RM	Kendall’s Tau = 0.205; *p* = 0.367	Valor z = 3.337; *p* < 0.001

**Table 5:** Results of subgroup analysis based on sampling region.

Subtype	Region	No. of Reports	Seroprevalence % (IC95%), *p*	Model (I^2^)	No. of Reports	Prevalence % (IC95%), *p*	Model, (I^2^)
H3N8	North America	11	7.96 (1.71–18.0), *p* < 0.001	RE, 99.3%			
	Europe	3	0.379 (0.00875–1.57), *p* = 0.006	RE, 99.7%			
H3N2	Eastern Asia	15	7.97 (4.98–12.2), *p* < 0.001	RE, 98.4%	9	16.2 (2.60, 38.4) *p* < 0.001	RE, 99.74%
	North America	4	6.86 (0.930, 18.0), *p* < 0.001	RE, 97.13	4	20.4 (6.94, 39.5) *p* < 0.001	RE, 98.7%
H1N1	Europe	5	1.31 (0.00009, 4.23), *p* = 0.010	RE, 96.05%			
	Eastern Asia	6	5.13 (0.741, 12.4), *p* < 0.001	RE, 98.99%			

## Data Availability

The data related of this study are available on request from the corresponding author.

## Supplementary Materials

The following supporting information can be downloaded at: https://www.mdpi.com/article/10.3390/ani14233467/s1, Supplementary Materials Figure S1: Sensitivity analysis graphs of the included studies. (a) H3N8 seroprevalence, (b) H3N2 seroprevalence, (c) H1N1 seroprevalence, (d) H3N2 prevalence, Supplementary Materials Figure S2; Funnel plot (a) for the analysis of H3N8 prevalence in dogs. (b) for the analysis of H3N2 prevalence in dogs.
