# Supplementary material for: Meta-Analysis of Seroprevalence and Prevalence of Influenza A Viruses (Subtypes H3N2, H3N8, and H1N1) in Dogs
Source: Animals (Basel). 2024 Dec 1;14(23):3467. doi: 10.3390/ani14233467 (PMC11640467; doi:10.3390/ani14233467)
Supplement: Supplementary file 1 [file animals-14-03467-s001.zip › animals-3266740-supplementary.pdf]

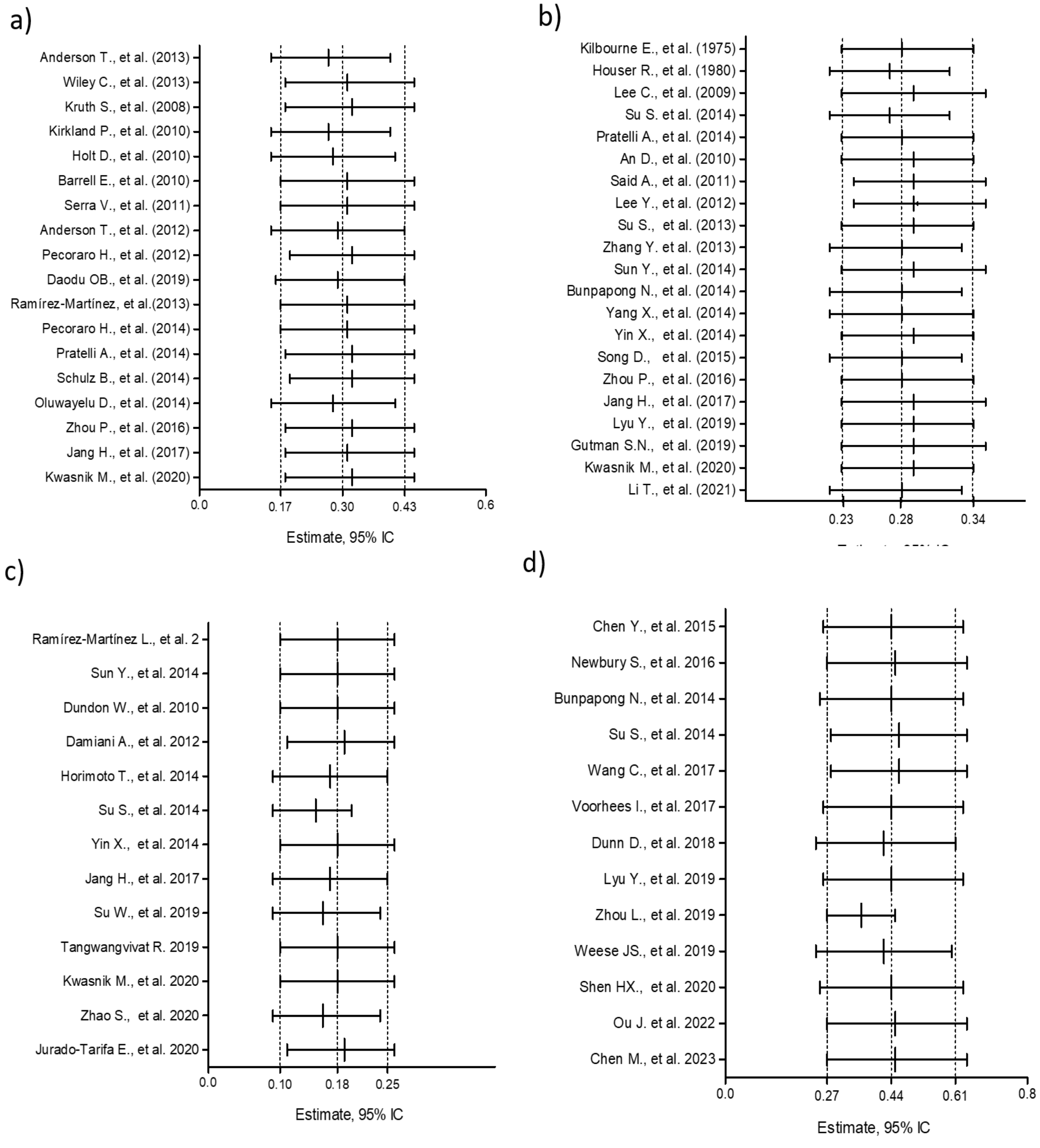

Figure S1: Sensitivity analysis graphs of the included studies. a) H3N8 seroprevalence, b) H3N2 seroprevalence, c) H1N1 seroprevalence, d) H3N2 prevalence

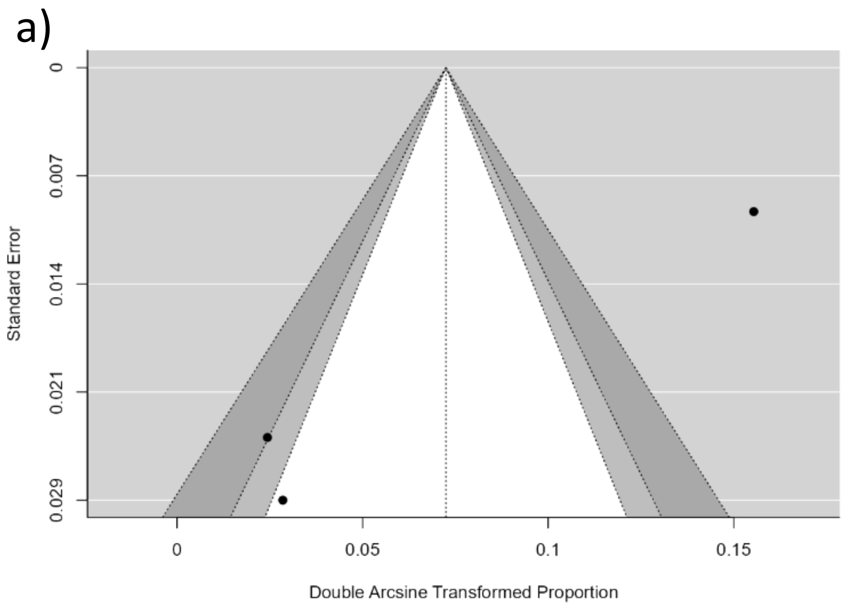

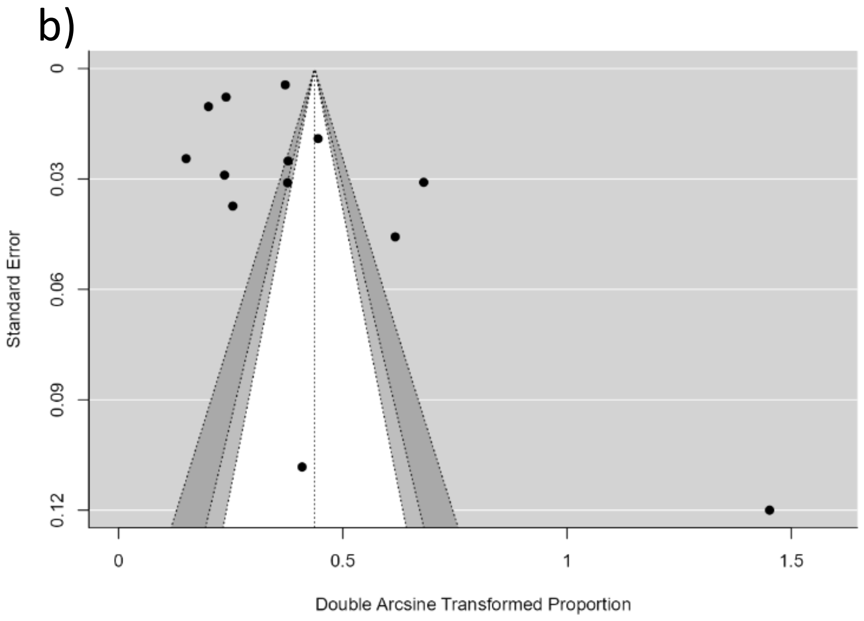

Figure S2; Funnel plot a) for the analysis of H3N8 prevalence in dogs. b) for the analysis of H3N2 prevalence in dogs
